# Enhancing listening skills among future public health professionals: a pre-post educational intervention study

**DOI:** 10.3389/fpubh.2025.1637788

**Published:** 2025-11-20

**Authors:** Kyle A. Kercher, Kathleen N. Heeter, Claire Dannelley, Julianna F. King, Samantha Schaefer, Vanessa M. Martinez Kercher

**Affiliations:** 1 Department of Kinesiology, School of Public Health-Bloomington, Indiana University, Bloomington, IN, United States; 2 Department of Applied Health Science, School of Public Health-Bloomington, Indiana University, Bloomington, IN, United States; 3 Department of Psychological and Brain Sciences, College of Arts and Sciences, Indiana University, Bloomington, IN, United States; 4 Healthy IU (Workplace Wellness Program), Indiana University Human Resources, Bloomington, IN, United States; 5 Department of Health & Wellness Design, School of Public Health-Bloomington, Indiana University, Bloomington, IN, United States

**Keywords:** wellness coaching, young adults, active listening, health and wellness coaching, college and university students

## Abstract

**Background/objective:**

The purpose of this study was to promote active listening skills among college-students majoring in health professions. The primary objective of this pre-post educational intervention study was to examine how students’ confidence in active listening skills changed during a 13-week wellness coaching training course. The secondary objective of the study was to examine how students’ baseline active listening skill confidence was correlated with change in active listening confidence across the course.

**Methods:**

This single-group pre-post design evaluated was used to evaluate listening skills among 74 college-students enrolled in a 13-week course. The service-learning component of the course was in collaboration with the workplace wellness program utilized by university employees. College-student participants engaged in 5-weeks of curricular training prior to being paired with university faculty and staff/employees who served as clients. Pre-to-post training changes in students’ confidence and knowledge in the provision of active-listening and behavior change support were evaluated. Three subscales were used to assess listening (AELS; sensing, processing, and responding) and an adapted Communication Evaluation in Rehabilitation Tool.

**Results:**

College students’ processing and reflective listening skills improved from pre- to post-intervention (*p < 0.01, p < 0.01*). Additionally, sensing, the most complex level of listening, also improved from pre- to post-intervention (*p* < 0.1). Lower baseline confidence in reflective listening was associated with greater improvements in active listening skills.

**Conclusion:**

Training undergraduate students in active listening represents a promising and feasible approach for enhancing communication skills within behavior-change education. Future studies should build on these findings by incorporating objective assessments of listening skill application to further strengthen evidence for this educational approach.

## Introduction

Listening is a critical skill for developing competent health professionals. While it may seem like a skill many people feel competent in, there is less understanding and awareness of the different types of listening and how these skills are integrated into daily life. There are many aspects of listening that require knowledge, skills, practice, and intentional integration into life and work. There are two different forms of listening individuals can engage in: active or passive. Active listening is defined as a type of communication that requires the communicator and listener to be fully present and engaged in a reciprocal conversation. An individual can improve this active skill in a variety of ways, including maintaining eye contact, actively engaging in conversation, asking questions, and more. While it may be known that active listening benefits individuals’ lives personally and professionally, it is not a skill everyone possesses.

In health professions (e.g., medical doctor, physical therapist, personal trainer, dietician, etc.), active listening is one of the most fundamental aspects to providing optimal care to patients/clients by understanding their concerns, perspectives, and symptoms while enhancing understanding (1, 2). When healthcare providers communicate with their patients and employ active listening, they can help identify what is known and what needs to be asked to develop care plans. Providers can ask follow-up questions to patients, leading to more engagement, rapport building, better healthcare outcomes, and ultimately a more accurate diagnosis with an individualized treatment plan.

Previous research has found that listening in healthcare and public health settings is vital to patients’ health and happiness (3). When patients do not feel heard, they report worse healthcare outcomes due to damaged rapport and misunderstandings about their needs and concerns (4). Conversely, when individuals feel heard in healthcare settings, they report more positive effects. Current research indicates that the perception of active listening activates reward pathways and leads to strengthened connections (5, 6). Research conducted on this skill found that individuals who felt heard reported a deep sense of connection through reciprocal communication whereas individuals who did not feel heard felt disregarded due to lack of listener engagement (7). Similarly, individuals who did not feel heard reported more negative psychological and social effects and indicated lower levels of connection and value in their profession (8, 9).

To complicate the context of listening, many people communicate primarily through electronic communications such as social media, text, and e-mail. While conversing virtually is often convenient and accessible, it may contribute to a lack of or deterioration of active listening kills (10). Technology and social media have cultivated a population who communicate virtually rather than in person (11). Individuals communicating through social media, email, and text messaging tend to disengage from conversations and become distracted by their external environments (12). Accordingly, listening is important in varied settings, but what exactly differentiates each type of listening, and how can one improve their listening skills?

Active listening goes beyond verbal language by integrating non-verbal language (i.e., body language). Active listening is a part of a broader form of learning called action learning, which is shown to enhance engagement and self-efficacy in learners (13). When using active listening as a form of active learning, individuals who are engaged in a conversation will actively show interest in what the speaker is saying through their body language, nodding their head, or maintaining eye contact (14). Active listeners engage speakers by asking questions about the topics they are discussing. Comparatively, passive listening is a form of listening in which the listener does not engage, verbally or non-verbally, with the speaker. This passive approach to listening can result in misunderstandings, as well as eroded connection, trust, and rapport between the speaker and listener (15). Notably, not all aspects of passive listening are detrimental, specifically when used in educational settings (16). For instance, students in the classroom often engage in passive listening where they are still actively engaging in the speaker’s communication, yet they are not engaging conversationally. This focus on passive listening is usually discussed alongside broader student skills like study habits and test taking strategies. The lack of discussion of active listening skills and reciprocal conversation can result in lackluster class participation and a miserable experience. Many students intend to go on to health professions, where communication most commonly occurs at a group level, thus making the need for teaching and improving active listening vital. Even within a more applied space, this skill is important to garner. Trained health and wellness coaches gain knowledge and skills in relationship establishment, motivational interviewing, perceptive reflections, wellness vision creation, and self-determined goal setting. Specific to listening, coaches also develop skills such as appropriate eye contact, mindful listening, and open body language to establish a supportive relationship with clients (17). It is particularly important for coaches to be proficient in listening as this skill is a part of communication competence, which allows them to exhibit proficiency and competence in their client work (18). Preexisting literature on active listening exists (7, 19, 20), but gaps in the literature remain within the types of populations studied (e.g., health professionals) and the impact and types of training.

Therefore, the purpose of this study was to investigate the impact of a service-learning course designed to promote active - preparing for careers in public health and health sciences. The course integrated behavioral science principles and health and wellness coaching techniques to strengthen communication and listening competencies that are essential to public health practice. The primary objective of this pre-post educational intervention study was to examine changes in students’ confidence in active listening skills during a 13-week wellness coaching training course. The secondary objective was to assess how students’ baseline confidence related to changes across the intervention. We hypothesized that students would demonstrate improvements in their listening skills. Results from this study have the potential to inform health professions education, training, and application by emphasizing active listening as a foundational communication skill.

## Methods

### Overview

**Figure 1:**
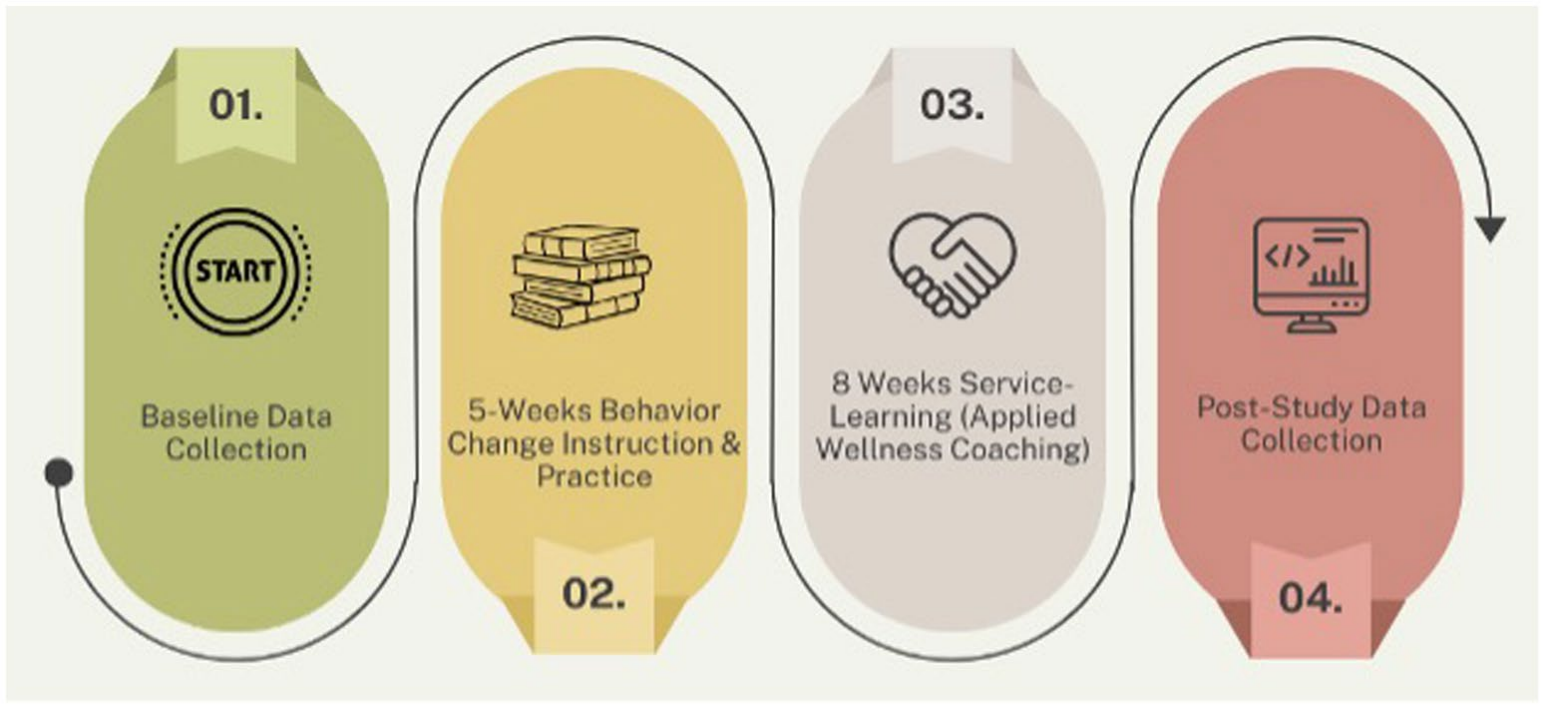
Study overview.

This pre-post educational intervention study with a evaluated the listening skills of college students (*n* = 74) enrolled in a 13-week wellness coaching training course. This pragmatic study was conducted in collaboration with an existing university employee workplace wellness program. College student participants underwent five-weeks of curriculum-based training prior to being paired with university faculty and staff who volunteered served as clients. Aside from a program orientation, these clients did not receive training and behaved as they normally would. Assessments were conducted pre- and post-training within the course. All students enrolled in the course participated in the study. See Figure 1 for a visual of the study overview.

### Setting and sample

The study was conducted with college students from public health majors enrolled in an academic class in the Department of Kinesiology at a Midwestern university. Inclusion Criteria for student coaches: (1) Over age of 18 years; (2) currently enrolled student in the Kinesiology course for majors in physical activity, fitness, and/or wellness; (3) willing to participate in the study. Exclusion criteria were: (1) not completing a consent form, and (2) not agreeing to participate in the study. No formal *a priori* sample size calculation was performed, as all students enrolled in the course during the semester were included in the study. A research assistant collected and anonymized all participant data prior to data analysis.

### Procedure

The course was delivered by a certified health and wellness coach (Wellcoaches^®^) with a doctoral degree and research background in sport and exercise psychology, measurement and evaluation, and behavior change interventions. The behavior change components were based on 21 motivation behavior change techniques aligned with self-determination theory communication strategies, wellness coaching principles, and other models and theories that support physical activity engagement (21). This included motivational interviewing strategies, client-centered verbal (e.g., expressing empathy, listening, reflections) as well as non-verbal (e.g., making eye contact) communication, and strategies to support basic psychological needs aligned with self-determination theory (e.g., competence, autonomy, and relatedness). Students were given opportunities to conduct role-play implementing the strategies and allowing practice of the skills relevant to wellness coaching (see Supplementary File 1). The 13-week course was divided into a training phase (Weeks 1–5) and a practice phase (Weeks 6–13). During the training phase, students received approximately 10 instructor-led sessions (75 min each; ~12.5 h total) focused on developing active-listening skills and coaching competencies. Training emphasized five key listening strategies—paraphrasing, reflective listening, summarizing, use of silence, and verbal acknowledgments—taught through the three levels of listening framework: sensing, processing, and responding. Instruction combined short lectures, modeling, and structured role-play exercises (e.g., ‘Listening Matters!’) with immediate peer and instructor feedback emphasizing empathy, reflection accuracy, and nonverbal presence. During the practice phase, students applied these skills in eight client sessions (first coach session at 55–60 min; seven coach sessions at 25–30 min/each) under weekly instructor supervision through check-ins, reflective journals, and debriefs. Further details are available in Supplementary Files 1–3. A social constructivist view point was utilized similar to other training approaches (22) where the instructor acted as facilitators promoting peer interaction and collaboration. Students were asked to work with peers in and outside of the classroom to create authentic and meaningful learning experiences they could personally connect to (23–25).

A brief outline of the course and summary of training and practice phases are provided in Tables 1, 2 (see Supplementary File 1 for additional information):

**Table 1:** Brief course outline.

Week	Brief outline of course
1	Course & study introduction, consent, pre-training survey
	Behavior change training begins
2–4	Behavior change training
5	Behavior change training, practical assessment, & student reflection
	Ready to move orientation; client + student meet & greet
6–13	Ready to move student & client weekly interaction
	Deeper knowledge: behavior change training
	Post-client feedback on student interactions (Week 13)
	Post-training survey
14–15	Student presentations on behavior change experience

Classes are held twice per week for approximately 75-min each.

**Table 2:** Training and practice phases summary.

Phase/Week (s)	Focus	Key listening strategies	Feedback and supervision	Estimated duration
Training (Weeks 1–5)	Core instruction in active-listening and coaching communication	Paraphrasing, reflective listening, use of silence, summarizing, acknowledgments	Instructor modeling, role-play feedback, peer discussion	~10 sessions × 75 min (~12.5 h)
Practice (Weeks 6–13)	Application of learned skills with clients (Ready to Move program)	Integration of previously taught strategies during real coaching sessions	Weekly instructor check-ins, reflective journals, group debriefs	8 sessions × 25–60 min
Wrap-Up (Weeks 14–15)	Reflection and synthesis of learning	Self-assessment, empathy evaluation	Peer review and instructor debrief	2 sessions × 75 min

**Figure 2:**
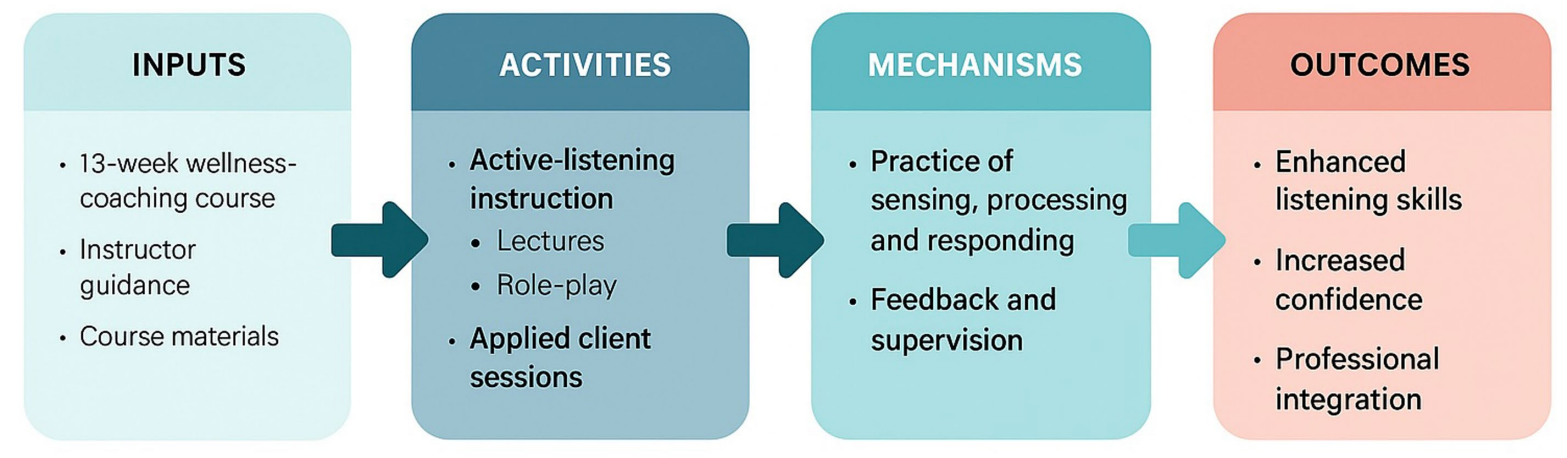
Logic model.

To illustrate the conceptual alignment among the course structure, learning processes, and expected outcomes, a logic model was developed and is presented in Figure 2. This model visually summarizes how the instructional design and applied learning components were structured to promote the development and application of active listening skills within public health contexts.

#### Recruitment

A course announcement was delivered to enrolled students by a research assistant with no prior relationship to students to minimize potential coercion. The purpose of the study was expressed, and students were able opt-in or opt-out of being in the study.

#### Student data collection

Two data collection points for the student coaches (e.g., pre-initial and post-exit). All students that provided consent were asked to complete a 7–8-min survey via Qualtrics (Qualtrics, Provo, UT) to assess study outcomes measures.

### Measures

The measures used for this study include demographics, behavior change components based on communication strategies within basic psychological needs within self-determination theory, and listening variables detailed below. Adopting a similar approach to a recent study with exercise science student majors (22), the effectiveness of the program was assessed using the Kirkpatrick model of evaluation at the levels of learning (26, 27). Learning explores the modification of learner’s knowledge, confidence, and increase in their skills. The measurement tools are described in detail below.

#### Demographics

Age, class standing, gender, and previous coaching experience were collected for each participant.

#### Listening

The Active-Empathetic Listening Scale (AELS) (28–30) was used to measure listening, and is comprised of three subscales: *sensing* (4-items; e.g., “I listen for more than the spoken word”), *processing* (3-items; e.g., “I keep track of points others make”), and *responding* (4-items; e.g., “I assure others that I am listening by using verbal acknowledgements”). Participants are asked to indicate how frequently they perceive each of the 11 statements to be true of themselves on a 7-point scale (1 = Never or almost true, 4 = Occasionally true, and 7 = Almost or almost always true). Individuals scoring higher in trait AELS have been found to make sharper distinctions between situations that varied in their putative need for activity and empathy (7, 30, 31). The reliability and validity of the AELS have been reported in various populations (Cronbach alpha = 0.94; ranging from 0.66–0.89 for each subscale) (30).

#### Motivation behavior change techniques (MBCT)

BCTs refer to observable, replicable, and irreducible component of an intervention designed to alter or redirect causal processes that regulate behavior; that is, a technique proposed to be an “active ingredient” (e.g., feedback, self-monitoring, and reinforcement) (32). BCTs can be used alone or in a combination and in a variety of formats and are effective to use for interventions to increase physical activity and changing professional behavior (32, 33). An adapted version of the Communication Evaluation in Rehabilitation Tool (CERT) (22) was used to measure learning and competency in communication skills utilizing BCTs (see Supplementary File 1). Student’s confidence in applying BCT components was assessed with scores ranging from 1 “not at all confident” to 7 “very confident” in line with previous work in public health trainees (22). Students were asked to indicate their degree of confidence in applying a specific BCT (see Supplementary File 1). The three BCT’s specifically focused on listening were included in this study and included: BCT 3. *Acknowledgement* referring to confidence to “acknowledge a client’s feelings and perspectives; BCT 9. *Reflective Listening* to report confidence to “use reflective listening skills”; and BCT 11. *Use of Silence* to measure confidence in one’s ability to “use silence regularly with a client.” This approach was used because it has demonstrated adequate reliability and validity and has been used in similar contexts (22, 34).

### Data analysis

All data analysis was conducted using RStudio (R 3.6.0+). Normality of continuous variables was assessed using the Shapiro–Wilk test. Screening for missing data, outliers, and normality was performed. Descriptive statistics with means, standard deviations, and bivariate correlations were calculated for all continuous variables. Less than 3% of data used for analysis was missing at random, therefore no data was imputed. Paired sample t-test were used to assess changes in listening from pre-study to post-study. All three subscales, processing, responding, and sensing were individually examined. To examine the relationship between changes in AELS processing and responding, we used subscale scores and baseline characteristics (confidence in BCTs and age) in a bivariate correlational analysis. Finally, to simplify the regression models, step backwards linear regressions were used. We assessed how student status at baseline affected change in processing and responding subscales of AELS and the impact of these characteristics on change, with confidence in BCT, age, and previous coaching experience as predictor variables.

## Results

Table 1 shows descriptive statistics for students at the baseline for the final sample of participants (*n* = 74). Participants were primarily undergraduate students (86.5% total, 21.6% sophomore, 28.4% Junior, 36.5% Senior) with 67.6% reported having had previous experience in coaching, and representative of females (51.4%) and males (48.6%). Participants’ results indicated increased confidence in BCT’s acknowledgment, reflective listening, and using silence after being enrolled in the course (see Table 3.)

**Table 3:** Descriptive statistics of continuous variables and correlations.

Variable	Mean	SD	Median [Min, Max]
Confidence in BCT			
*Acknowledgement*	5.51	1.310	6.00 [1.00, 7.00]
*Reflective Listening*	4.65	1.340	5.00 [1.00, 7.00]
*Use Silence*	3.97	1.440	4.00 [1.00, 7.00]
Age (Years)	21.6	3.330	21.0 [19.0, 40.0]

### Changes in listening

Results indicated a significant and positive change in processing, responding, and sensing subscales between pre-study and post-study listening scores. Mean values of subscales, changes in subscales, and delta estimates from t-tests are shown in Table 4.

**Table 4:** Changes in active listening skills.

Active Empathetic Listening Scale (AELS)	Pre-study mean (SD)	Post-study mean (SD)	Change mean (Delta Estimate)
Processing	5.00 (0.937)	6.02 (0.733)	1.030 (−1.165)**
Responding	5.66 (0.815)	6.35 (0.563)	0.711 (−1.208)**
Sensing	4.81 (0.879)	5.76 (0.742)	0.965 (−1.237)**

** = *p* < 0.01 on paired sample t-test.

### Correlations to change

Bivariate correlations between changes in AELS subscales and baseline continuous descriptive statistics (BCTs and Age) are shown in Table 5. Participants’ confidence in acknowledgement and reflective listening was significantly associated with all three AELS subscales. Using silence was significantly associated with processing and responding subscales. Age was not associated with any AELS subscales.

**Table 5:** Correlations to change in AELS scores.

Baseline measure	AELS Subscale		
	Process	Respond	Sensing
Confidence in BCT			
*Acknowledgement*	−0.273*	−0.295*	−0.256*
*Reflective Listening*	−0.383**	−0.480**	−0.345
*Use Silence*	−0.279*	−0.307**	−0.032
Age (years)	0.038	0.041	−0.082

* = *p* < 0.05, ** = *p* < 0.01.

### Participant growth trends

Step backwards linear regressions for all AELS subscales are provided in Table 6. In all three models, results indicated that confidence in BCT Reflective Listening skills (e.g., “use reflective listening skills”) was a significant negative predictor of changes in AELS subscales. This implies that higher BCT Reflective Listening scores at baseline predict smaller changes in AELS subscales throughout the study. Adjusted R-squared values for the processing, responding, and sensing models are 0.163, 0.234, and 0.108, respectively. The tests for normality, independence, and multicollinearity found that in all three models’ data was normal, independent, and there was no multicollinearity.

**Table 6:** Step backwards regression models.

Variables	Beta	SE	*t*-value	*p*-value
AELS processing subscale				
Intercept	3.192	0.565	5.648	<0.01
BCT *Acknowledgement*	−0.101	0.092	−1.104	0.274
BCT *Reflective Listening*	−0.200	0.091	−2.191	0.032
BCT *Use Silence*	−0.119	0.076	−1.564	0.123
Coaching experience (Yes/No)	−0.296	0.224	−1.327	0.189
AELS responding subscale				
Intercept	2.322	0.346	6.711	<0.01
BCT *Reflective Listening*	−0.263	0.068	−3.851	<0.01
BCT *Use Silence*	−0.096	0.062	−1.549	0.126
AELS sensing subscale				
Intercept	2.317	0.46	5.018	<0.01
BCT *Acknowledgement*	−0.091	0.083	−1.088	0.280
BCT *Use Silence*	−0.183	0.079	−2.317	<0.05

## Discussion

The current pre-post educational intervention study examined changes in listening skills among college students enrolled in a 13-week wellness coaching training course. The study examined the relationship between three behavior change techniques and three levels of listening. There were 3 key findings. First, college students processing and reflective listening skills, the two more rudimentary levels of listening skills, improved from pre- to post-intervention. Second, sensing, which is the most complex level of listening, also showed significant improvement. Third, students with the lowest confidence in reflective listening experienced the largest changes across the intervention. As the field of wellness coaching continues to grow, the findings from this educational intervention study may help inform future wellness coaching-related research examining coaching methodology to improve listening in more rigorous study designs.

First, the intervention results showed that two of the subscales of the AELS, processing and responding, improved from pre- to post-intervention. This result indicated that, participants had better processing and responding listening skills after participating in the training course. Furthermore, this outcome may indicate that these processing and responding listening skills can be acquired or improved upon in a relatively short period of time. In this case, it exemplifies that a 13-week course (i.e., 5 weeks of training, 8 weeks of practical application) focused on behavior change techniques and listening appears to meet the required dose needed to illicit such an association. As such, these findings support the inclusion of listening curricula, such as modules and readings on active listening and empathetic communication, into health-professional education. Second, the most cognitively complex level of listening, called sensing, also significantly improved from pre- to post-intervention. There are several potential reasons this improvement may have occurred. Of note, there has been limited previous research on changes in listening skills of coaches’ or other health professionals. A study conducted by Ickes and McMullen (35) among future health promotion professionals found that following a campus-based training course, participants felt more confident in their health coaching skills (i.e., motivational interviewing, reflective listening, openly communicating). A systematic review conducted by Parry (36) found that there was little direct evidence or recommendations regarding curriculum to help improve communication skills in allied health professionals, such as wellness coaches. In the present study, the training phase of the course lasted five-weeks with two sessions per week. This structure may have provided sufficient exposure for students to better understand and apply the cognitively complex task of sensing within the wellness-coaching training process. Additionally, the college students in the course were relatively young with a mean age of 21.6 years old. Individuals at this age range may still be developing empathy and perspective-taking skills that contribute to higher-level sensing abilities. The results suggest that even limited but structured exposure to active-listening practice may support development across all three levels of listening. Future studies could explore whether longer training duration or more advanced practical experiences further enhance these higher-order listening skills in diverse student populations. Third, students that had lower confidence in the behavior change technique of “using reflective listening” at baseline experienced the greatest change in both the processing and responding subscales of the AELS. While there may be multiple explanations for this finding, one explanation is that the AELS processing subscale and the AELS responding subscale are correlated to a student’s confidence in their reflective listening skills. It is likely that students who have high confidence in their reflective listening skills at baseline, will also self-report their active listening skills as high at baseline. Students who have already scored themselves high at the baseline of the course/study will tangibly have less room for psychometric scale improvement throughout the study.

Finally, the above findings highlight the potential for incorporating behavior change training in coach training courses to support long-term development of progressive coach-client relationships. Coaching certification courses, both in person and online, have been on the rise, leading to discussion of what information should be within the courses (37). An important aspect of coaching is the ability of coaches to support behavior change in clients. Previous research recognizes that having empathetic listening skills bolsters the coach-client relationship (38, 39). This potentially serves as an argument for the incorporation of behavior change theory teaching in coaching courses to increase empathetic listening skills, and thus further support the coach-client relationship. Just as coach-client relationships can benefit from improved listening skills, empathetic listening skills may also be learned in other settings throughout one’s life. Building empathy may also support sensing skills. A notable strategy to support empathy development is through socially oriented approaches that focus on enhancing social skills and enjoying positive social interaction (e.g., peer support, theoretical or practical communication skills training) (40).

In addition, while training students in active listening represents a promising educational strategy within behavior-change curricula, the current findings are based on self-reported confidence rather than direct observation of listening behavior. Future investigations should incorporate objective or performance-based assessments (e.g., structured observation, peer-rating, or simulated-client evaluations) to evaluate skill acquisition and application more comprehensively. From an educational perspective, these findings also align with broader frameworks of competency-based learning and reflective practice, which emphasize iterative cycles of practice, feedback, and self-assessment as essential to developing professional communication competence. Embedding these frameworks into wellness-coaching and public-health education may further strengthen students’ readiness to engage in client-centered communication and behavior-change support.

The results of this study must be considered within its limitations. First, the small sample size (*n* = 74) limits the types of analyses that could be conducted and reduces how broadly the findings can be generalized. Second, because this was a single-group pre-post design without a control or comparison group, the results should be interpreted with caution. Third, the data relied on students’ self-reported confidence in their listening skills rather than on observed behavior, which may overestimate actual improvement. No formal sample-size calculation was conducted, as all students enrolled in the course were included in the study. Taken together, these findings should be viewed as exploratory. Future research with larger samples, objective or observational measures, and control groups could help build on this work and deepen understanding of how active-listening training impacts public-health and wellness education.

This study, to our knowledge, is one of the first that examines how listening skills change following participation in a wellness coaching training course, or what predicted these changes. Processing, responding, and sensing listening skills all improved throughout the study, suggesting that structured training and applied practice may support development across multiple levels of active listening. Additionally, the results indicated that students with high confidence in reflective listening skills at baseline had smaller changes in AELS processing and reflective AELS subscales. This may be explained that if students are already confident in their listening skills, they are likely to have high baseline AELS scores. These overall findings highlight the importance of embedding structured listening and behavior-change training into public-health and wellness-coaching education to better prepare future professionals for client-centered communication and behavior-change support.

## Data Availability

The raw data supporting the conclusions of this article will be made available by the authors, without undue reservation.
