# Supplementary material for: Enhancing listening skills among future public health professionals: a pre-post educational intervention study
Source: Front Public Health. 2025 Nov 20;13:1637788. doi: 10.3389/fpubh.2025.1637788 (PMC12677012; doi:10.3389/fpubh.2025.1637788)
Supplement: Supplementary file 1 [file Supplementary_file_1.docx]

**Supplement 1. Coach Training Protocol**

## **
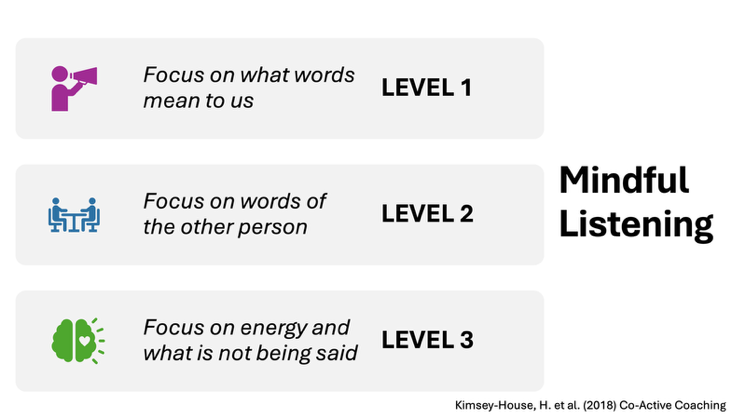

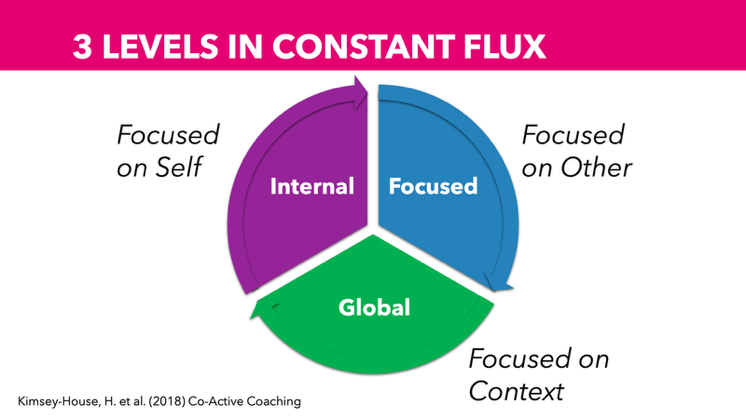
Training (Weeks 1-5).** The first five-weeks of the course was the introduction of core-coaching competencies, behavior-change related theories, strategies, and skills through formal didactic in-class sessions, lectures, video-lectures, simulated patient encounter audio/video recording samples for students to listen to, as well as individual exercises and activities with supplemental lecture materials. Further, interactive in-class activities and assignments were developed to help students practice applying behavior-change techniques through role and real-play with classmates, self-reflection, and self-assessment of competence to facilitate ongoing skill development prior to working with client. A detailed example of activities completed focused on listening based on strategies utilized within coaching and other sources (1) can be found in figures below.

**Ready to Move: Practical Application (Weeks 6-13):** After five weeks of course lectures, exercises, activities, each student was individually and randomly paired to work with a university employee (i.e., client) enrolled in a program, Ready to Move. Each student was required to meet with the client eight times throughout the semester outside of the classroom for an average of 25-30 minutes via zoom or phone except for the first coaching session (55-60 minutes) which was longer in duration. A coach tracking sheet with parameters was developed by each student coach covered in course material focused on program goals, weekly goals, motivators, barriers, confidence, time spent (e.g., session date and mode), and tools/resources provided to the client that students submitted weekly.

**Supplement 2. Listening Lessons & Exercises Sample**

The following course objectives focused on listening.

| **Course Learning Objectives** |
| --- |
| Understand the value of listening in communication |
| Describe important aspects of the three levels of listening |
| Identify important elements of nonverbal listening behaviors |
| Evaluate how verbal/ nonverbal listening behaviors impacts communication |
| Practice and apply listening skills |

**Note.* These learning objectives have been modified based on Schmidt (2018).

**Breakout 1: Listening Matters! Exercise**

| **Pre-First Interaction (5 Minutes)** |
| --- |
| *Instructions:* |
| Randomly break out students into pairs with one student playing the role of “Coach” and the other as “Client”. |
| Breakout students into their pairing and into zoom room. |
| Provide each student a role sheet for the first interaction topic, based on whether they are playing the role of the “coach” or “client”. The sheets include a topic for the client/speaker and role behaviors for each. |
| Encourage students to take about ~3-minutes to read the role sheet and prepare for the mock, role-play interaction. |

**Client Role:** The role of the clients is presented on their role sheet a subject to talk about with their coach, who is also viewed as the listener for a period of ~ 3-minutes. The topic used for this first interaction was to “*describe a time when you tried to change a behavior in the past*” that resulted in a successful or unsuccessful outcome. The “clients” are instructed to share the information when asked and be able to select an area that would encourage a thoughtful response to their “coach”. In the role of the client, the student is asked to primarily focus on listening.

**Coach Role:** The coach can also be seen as the listener, and are given simple parameters on their behavior when acting in this role. Coaches are told not to ask any questions and remain silent while listening as best as possible. If the client asks the coach a question, the coach is instructed to give as short and noncommittal a response as possible. Additionally, during this role-play activity the coach is asked to a) pay attention to what thoughts take place in their head, b) write down thoughts that distract them from listening; and c) identify what level of listening (e.g., level 1, 2, or 3) they tend to adopt and/or exhibit within the listening exercise.

| **First Interaction (5 Minutes)** |
| --- |
| *Instructions:* |
| Pair each student who to play their role as the “coach” or “client”. |
| Breakout students into their paired zoom room |
| Provide students with opportunity to introduce each other and confirm their known role ~1-2 minutes. |
| The facilitator/instructor will send a broad message to all students in their zoom room when it’s time to begin and encourage the “coach” to set a ~3-minute timer as they begin. |
| If there is an odd number of students, the facilitator/instructor could act in a pair or have the person without a pair act as an observer for one pair or for the class as a whole. |
| The facilitator will also time the ~3-minute interaction and send a zoom announcement to all breakout rooms encouraging each student to stop and prepare to switch roles (~1-minute). |

| **Pre-Second Interaction (5 Minutes)** |
| --- |
| *Instructions:* |
| Have students stay in their pairs based on their role, but switch roles. |
| In the role switch—the coaches will now become the client; and the client will now become the coach. |
| Tell students to take to take a minute to re-read the role sheet and prepare for their role. |
| The facilitator/instructor will send a broad message to all students in their zoom room when it’s time to begin and encourage the “coach” to set a ~3-minute timer as they begin. |

| **Debriefing the First Interaction (5-7 Minutes)** |
| --- |
| *Instructions:* |
| Bring the whole class together in the zoom room to discuss the interaction. |
| **Coach Role:** First, encourage all students to reflect on their role as the coach. Ask questions of students and the group as a whole that include:   - “What distracted you from fully paying attention? - “What thoughts did you list as being a distraction to you? - “What levels of listening did you find yourself engaging/not engaging in? - “What else did you notice about your listening? |
| **Client Role:** Second, encourage all students to reflect on their role as the client. Ask questions of students and the group as a whole that include:   - “What was your experience as the client like? - “What did you like/dislike about the role? - “What else did you notice as you were sharing your behavior? |
| ***General comments:*** The class discussion elicits reactions and analysis from all students. Generally, in the classroom when students played the role of the clients, they often express that the interaction was awkward, they ran out of things to say, and not being used to talking for long periods of time without interacting with their partner/peer. When the students played the role of the coach, they often share how difficult it was to not ask questions during the interaction and how much they wanted to interject whether it be to express agreement or excitement about the subject because the coach also had a similar experience (e.g., level 1 listening). |

**Supplement 3. Self-Determination Theory based Communication Strategies.**

The table below represents student participants’ learning of delivery of a communication style based on self-determination theory (SDT) based communication strategies representing course activities and lessons in line with Texeira et al. (2019) supported by Deci & Ryan (2017), and also aligned within a modified version of the Communication in Rehabilitation Evaluation Tool (CERT).

| **Students’ Delivery of Communication style via SDT-based Strategies**  **Modified CERT Scale ^a^* | | |
| --- | --- | --- |
| **ITEM** | **Strategy** | **Description of strategy** |
| 1 | Open ended questions | The coach intentionally used open ended questions at the beginning of a discussion and where necessary closed-ended questions during follow-up questioning to obtain specific information. [Relatedness] |
| **2** | **Staying silent** | The coach encouraged the client to complete sentences and finish speaking before following up with further questions. [Relatedness] |
| 3 | Summaries | After listening to the client, the coach summarised his/her perception of what the client had said. The coach also may ask client with the opportunity to confirm or clarify. [Relatedness] |
| **4** | **Reflection ^b^** | The coach shows the client that he/she understood the client’s perspective by reflecting to the client what they said, either by simply repeating or slightly rephrasing what they had said or by making a guess as to what they may have meant. [Relatedness] |
| 5 | Asking permission | The coach asked the client if they were ready to consider advice regarding physical activity and/or the coach may have asked the client’s permission to give some information or advice. [Autonomy] |
| 6 | Provide meaningful rationale | The coach explained to the client the rationale behind his/her advice. [Autonomy] |
| 7 | Opportunities for client input/choice | The coach asked the client for their opinion/input about their behavior change. In doing so, the client was given an opportunity to contribute to the conversation in a meaningful way. [Autonomy] |
| 8 | Autonomy supportive language | The coach attempted to motivate the client by supporting their autonomy in the situation (e.g., by using supportive and encouraging and flexible language rather than using coercion or pressurizing behavior, or guilt inducing words/phrases). [Autonomy & Competence] |
| 9 | Goal-setting and action planning | Goals that are specific, measurable, achievable, and time-based were discussed by coach and client. These may not be named as “goals” in the conversation but are actions that the person is intending to carry out, and meet some or all of the SMART criteria. [Competence] |
| 10 | Barrier identification | The coach and client discussed a likely barrier (or barriers) to following advice or changing behavior.  [Autonomy & Competence] |
| 11 | Solution identification | The coach and client brainstormed about how they might overcome this barrier. [Autonomy & Competence] |

*Note.* SDT’s psychological needs represented within each item above are included in description as either [Autonomy, Competence, and/or Relatedness].

^a^ There were seven strategies listed in the Communication in Rehabilitation Evaluation Tool (CERT) that were excluded from this study. These items were: Use single questions; catering for different learning preferences; closing the loop; provision of a rehabilitation diary, contact details; and follow-up.

^b^ The strategy of empathy listed in the CERT was renamed and adapted to reflection.

**Supplement 4. Motivation-Behavior Change Techniques (BCT’s).**

Based on the work of Texeira et al. (2019) and the Communication in Rehabilitation Evaluation Tool (CERT) tool, the following items were used to assess learning of behavior-change techniques assessed pre and post course. Specifically, items #3, 9, and 11 that are highlighted and bolded below were specifically included because of their focus on listening which was the primary focus of this study. A total confidence score based on all participants responses to the items listed below were assessed using a Likert scale ranging from 1=“*not confident at all*” to 7=“*very confident*” detailed below.

| **Item** | **Motivation Behavior Change Component** |
| --- | --- |
| BCT 1 | How confident am I in my ability to explore a client's values and goals? |
| BCT 2 | How confident am I in my ability to use non-controlling language (e.g., "should", "must")? |
| **BCT 3** | ***How confident am I in my ability to acknowledge a client’s feelings and perspectives?** |
| BCT 4 | How confident am I in my ability to express positive support regardless of success or failure? |
| BCT 5 | How confident am I in my ability to use empathy with clients? |
| BCT 6 | How confident am I in my ability to assist a client to set SMART goals? |
| BCT 7 | How confident am I in my ability to address obstacles/barriers in a positive way? |
| BCT 8 | How confident am I in my ability to ask open-ended questions? |
| **BCT 9** | ***How confident am I in my ability to use reflective listening skills?** |
| BCT 10 | How confident am I in my ability to respond to client resistance? |
| **BCT 11** | ***How confident am I in my ability to use silence regularly with a client?** |

*Note.* Bold font and “*” represents listening-focused items reported in this study.
